# Development of “3D printing in medicine” course leads to creation of a functional prosthetic for underserved patient

**DOI:** 10.1002/pmrj.13427

**Published:** 2025-05-30

**Authors:** John Spencer Laue, Thomas Shevlin, Sarika Mullapudi, Peter Anthony, Conley Carr

**Affiliations:** ^1^ Equal Access Birmingham University of Alabama at Birmingham Birmingham Alabama USA; ^2^ Department of Physical Medicine and Rehabilitation University of Alabama at Birmingham Birmingham Alabama USA

At Equal Access Birmingham, a free community health care clinic run by medical students, patients without adequate access to medical care often seek care that is beyond our capabilities. A patient presented to the clinic with a unique transmetacarpal amputation from a crush injury with severe scarring and compaction of her palm. Prior to meeting this patient, we partnered with the Biomedical Engineering Department to create a “3D Printing in Medicine” co‐enrolled class. Throughout this course we learned the skills and techniques to design and construct medical devices. Using 3D printing technology, we aimed to provide the patient with a cost‐effective and functional prosthetic.

The patient's residual limb was scanned via Shining 3D Einstar Handheld scanner to provide an amputation model as seen in Figure 1A. The e‐NABLE K1 hand prosthetic model was selected as the base model due to its ability to flex and close the fingers using a wrist flexion‐based mechanism. The model was modified and reconstructed around the amputation using Fusion 360 computer‐aided design software. Because of the patient's uninjured fifth digit, the palm and finger sections were designed to wrap around the residual components allowing for a personalized fit as seen in Figure 1B. After initial production and testing, the design was altered to allow for a more natural distribution of the finger joints and enable greater use of the grasping mechanic as seen in Figure 1C. Padding was added to provide comfort to the patient.

**FIGURE 1:**
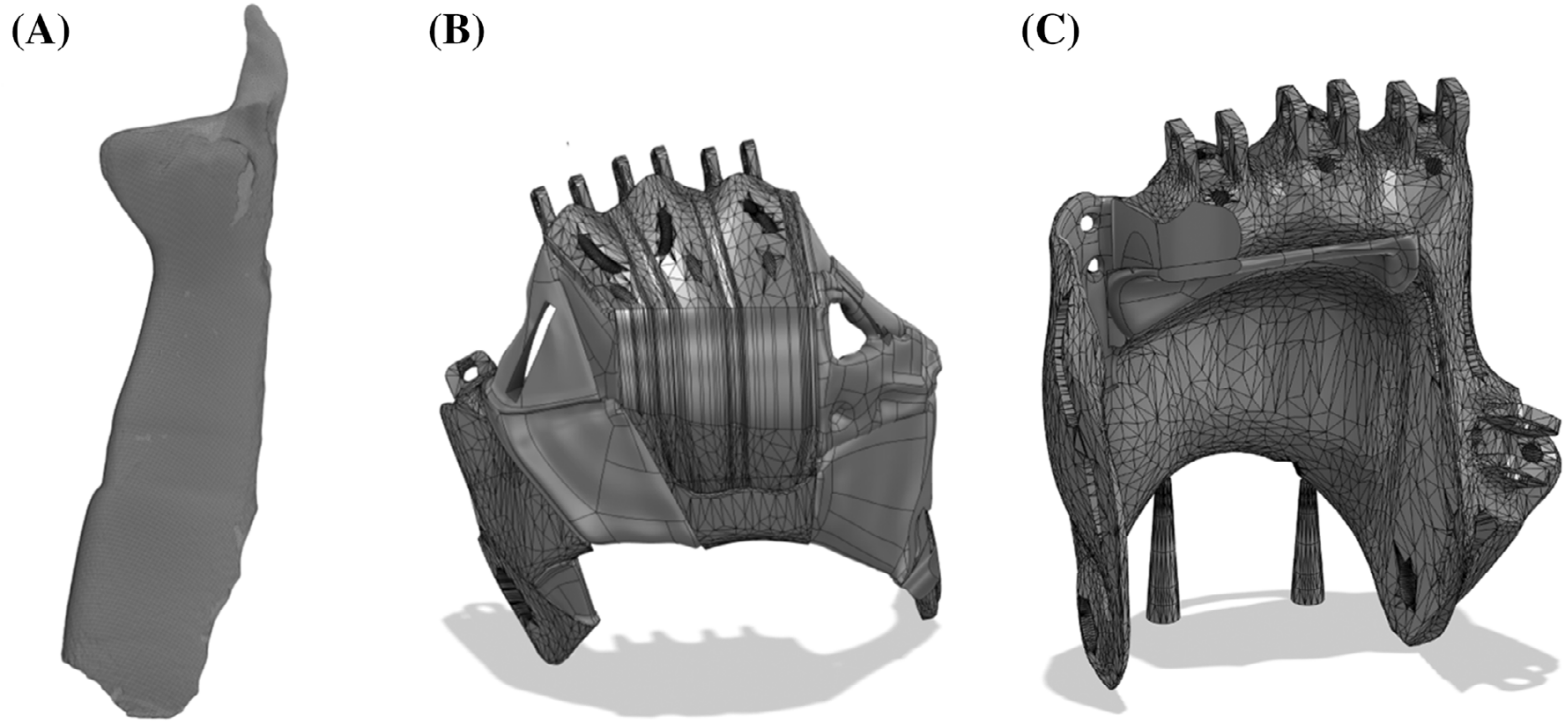
(A) 3D scan of patient's residual limb used to model prosthetic. (B) Original modification of e‐NABLE K1 hand prosthetic to match patient anatomy. (C) Revised modification of hand prosthetic.

Using patient feedback at the patient's following appointment, the prosthetic was scaled to match her other limb, the forearm was altered for a better fit, and the filament color was changed to better reflect her skin color. Because of a titanium rod in the patient's forearm, we reached out to the Orthotics and Prosthetics Department to help provide us with additional medical grade padding. The final product shown in Figure 2 was then brought back to the patient and the fit was evaluated to be comfortable, functional, and pleasing to the patient.

**FIGURE 2:**
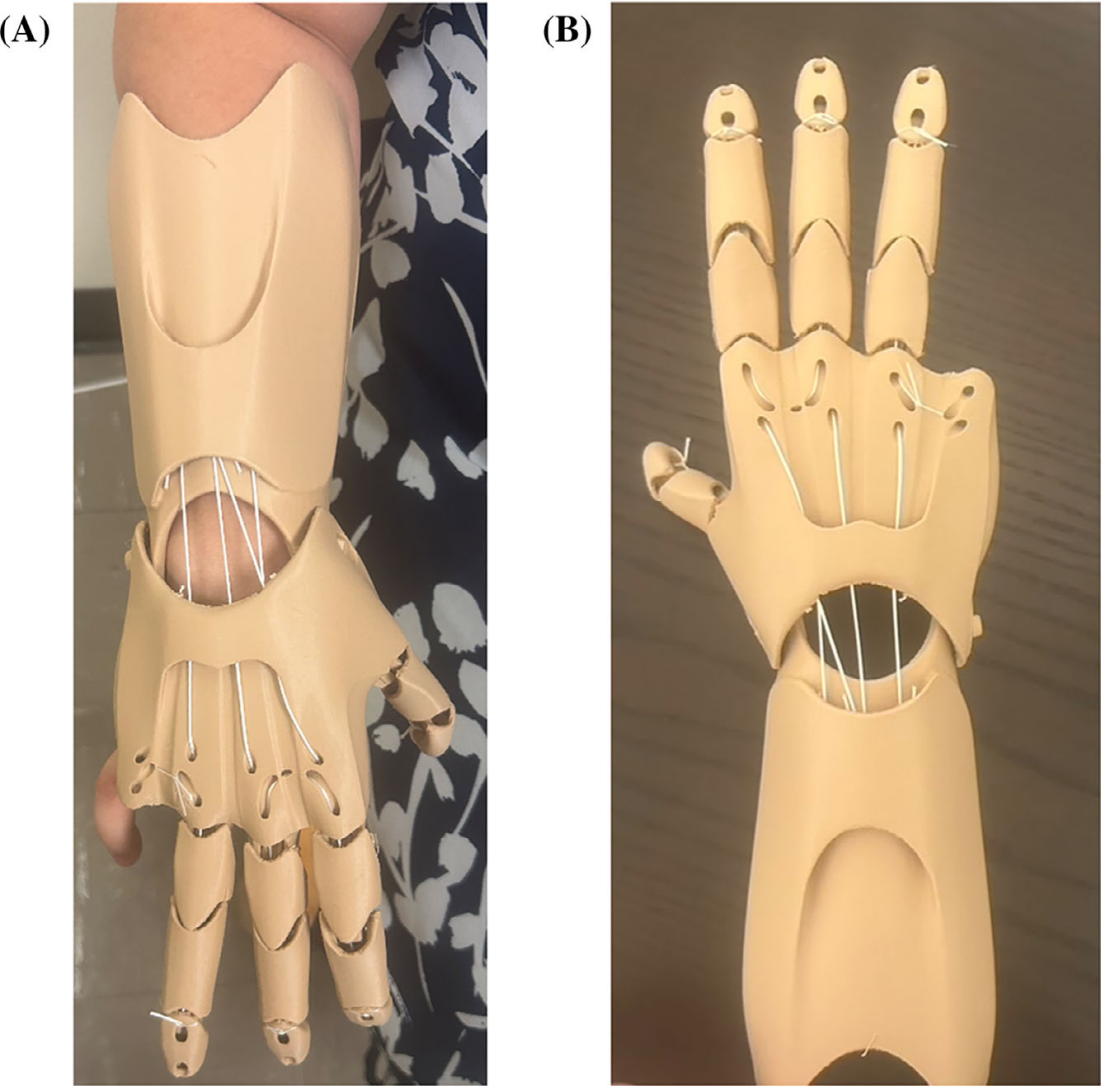
(A) Final prosthetic fitted on patient. (B) Final design shown after production.

Our work aligns with and builds upon existing research in 3D‐printed prosthetics, particularly in the domain of personalized and cost‐effective medical devices. As prosthetic prices rise with advancing technology, 3D printing offers a practical solution for those unable to afford costly options.^1^ Studies have demonstrated the efficacy of 3D printing in creating functional upper limb prostheses, highlighting its affordability, customizability, and rapid prototyping capabilities. For instance, research has emphasized the potential of open‐source designs, such as e‐NABLE models, to provide functional restoration at a fraction of the cost of traditional prostheses.^2^ Our approach similarly leveraged open‐source designs but was uniquely adapted to the patient's specific anatomy, demonstrating the adaptability of these models to more complex cases.

Compared to studies focused on standard transradial or transhumeral amputations, our case presented a significant challenge due to the patient's unique transmetacarpal amputation with extensive scarring. Prior research notes that individuals with partial amputations often face greater difficulties achieving optimal prosthetic fit due to anatomical variations and altered joint function.^3^ By using advanced scanning and computer‐aided design techniques, we addressed these challenges and refined the prosthetic to ensure a better anatomical fit, demonstrating the potential for patient‐specific modifications in 3D‐printed prosthetic solutions.

Our project also highlights the growing role of interdisciplinary collaboration in medical device innovation. Literature underscores the necessity of partnerships between health care professionals, engineers, and prosthetists to optimize outcomes.^4^ Our collaboration with the Biomedical Engineering and Orthotics and Prosthetics Departments exemplifies this, reinforcing the value of integrating medical expertise with engineering to develop tailored health care solutions.

Despite its successes, our work also highlights limitations associated with 3D‐printed prosthetics. Studies have noted material strength and durability as common concerns in additive manufacturing for medical use.^5^, ^6^ Although our design incorporated medical‐grade padding and structural modifications for improved comfort and function, long‐term durability remains an area for future improvement. Patient feedback also emphasized aesthetics, aligning with research showing that appearance plays a crucial role in prosthetic acceptance and use.^7^ Our adjustments in filament color and material reflect the need to consider psychosocial factors alongside function. For a device to succeed, its design must align with user expectations and effectively meet their needs. Many cases of abandonment stem from issues with functionality, reliability, appearance, comfort, and usability.^8^

Overall, our work contributes to the growing body of evidence supporting 3D printing as a viable, patient‐centered approach to prosthetic design. By combining open‐source models, advanced scanning techniques, and interdisciplinary collaboration, we developed a personalized, cost‐effective prosthetic for a patient with a complex amputation. Future efforts should focus on enhancing material properties, increasing durability, and expanding accessibility to similar solutions for underserved populations.

## DISCLOSURE

The authors declare that they have no known competing financial interests or personal relationships that could have appeared to influence the work reported in this manuscript.

## CONSENT

Written patient consent has been obtained for written and photographic works.
